# Persistence of Resident and Transplanted Genotypes of the Undomesticated Yeast Saccharomyces paradoxus in Forest Soil

**DOI:** 10.1128/mSphere.00211-18

**Published:** 2018-06-20

**Authors:** James B. Anderson, Dahlia Kasimer, Wenjing Xia, Nicolas C. H. Schröder, Patrick Cichowicz, Silvio Lioniello, Rudrakshi Chakrabarti, Eashwar Mohan, Linda M. Kohn

**Affiliations:** a Department of Biology, University of Toronto, Mississauga, Ontario, Canada; Carnegie Mellon University

**Keywords:** 5FOA resistance, dispersal, fungus, genetic drift, population

## Abstract

One might expect yeasts in soil to be highly dispersed via water or insects, forming ephemeral, genetically heterogeneous populations subject to competition and environmental stochasticity. Here, we report persistence of genotypes of the yeast Saccharomyces paradoxus in space and time. Within 1 km^2^ in a mixed hardwood forest on scales from centimeters to tens of meters, we detected persistence over 3 years of native genotypes, identified by single nucleotide polymorphisms (SNPs) genome-wide, of the wild yeast Saccharomyces paradoxus growing around Quercus rubra and Quercus alba. Yeasts were recovered by enrichment in ethanol-containing medium, which measures only presence or absence, not abundance. Additional transplantation experiments employed strains marked with spontaneous defects in the *URA3* gene, which also confer resistance to 5-fluoroorotic acid (5FOA). Plating soil suspensions from transplant sites on 5FOA-containing medium permitted one-step quantification of yeast CFU, with no interference from other unmarked yeasts or microorganisms. After an initial steep decrease in abundance, the yeast densities fluctuated over time, increasing in association with rainfall and decreasing in association with drought. After 18 months, the transplanted yeasts remained in place on the nine sites. *In vitro* transplantation experiments into nonsterile soil in petri dishes showed similar patterns of persistence and response to moisture and drought. To determine whether Saccharomyces cerevisiae, not previously recovered from soils regionally, can persist in our cold climate sites, we transplanted marked S. cerevisiae alone and in mixture with S. paradoxus in the fall of 2017. Five months later, S. cerevisiae persisted to the same extent as S. paradoxus.

**IMPORTANCE** *Saccharomyces* yeasts are intensively studied in biological research and in their domesticated roles in brewing and baking, and yet, remarkably little is known about their mode of life in forest soils. We report here that resident genotypes of the yeast S. paradoxus are persistent on a time scale of years in their microhabitats in forest soils. We also show that resident genotypes can be replaced by transplanted yeast genotypes. The high inoculum levels in experimental transplantations rapidly decreased over time, but the transplanted genotypes persisted at low abundance. We conclude that, in forest soils, *Saccharomyces* yeasts exist at very low abundance and that dispersal events are rare.

## Abstract (precis)

*Saccharomyces* yeasts are intensively studied in biological research and in their domesticated roles in brewing and baking, and yet, remarkably little is known about their mode of life in forest soils. We report here that resident genotypes of the yeast S. paradoxus are persistent on a time scale of years in their microhabitats in forest soils. We also show that resident genotypes can be replaced by transplanted yeast genotypes. The high inoculum levels in experimental transplantations rapidly decreased over time, but the transplanted genotypes persisted at low abundance. We conclude that, in forest soils, *Saccharomyces* yeasts exist at very low abundance and that dispersal events are rare.

## INTRODUCTION

In their review of the ecology and evolution of nondomesticated *Saccharomyces* species, Boynton and Greig (1) encouraged further investigation of these species in nature to place them in ecological context with reference to the scientific model Saccharomyces cerevisiae, a long-domesticated yeast for food and beverage fermentation. Our focus here was on Saccharomyces paradoxus, the most tractable comparator of wild to domesticated *Saccharomyces*. S. paradoxus is associated with, but not limited to, oak leaf litter in forest soils (2, 3). In this yeast, progress has been made on understanding the relative importance of dispersal, genetic drift, and local adaptation in populations (4) and the effects of substrate utilization on metabolism and fitness (5, 6). *Saccharomyces* yeasts are present in soil and plant material, especially bark of deciduous trees where they utilize exudates such as sap. Filteau et al. (6) have elucidated the genetic basis of a fitness determinant in S. paradoxus: degradation of allantoate, the major nitrogen source in maple sap. However, there has been little information on the absolute abundance of these yeasts in their natural habitat. Most information on the occurrence of wild yeasts in soil has come through enrichment culture in ethanol-containing medium, which cannot be used to estimate abundance. A major goal of this study was to measure the abundance over time of yeasts transplanted to their natural substrates at high initial densities.

Dispersal of *Saccharomyces* species between substrates is poorly understood (1), but the recent work of Boynton et al. (4) suggests that lack of dispersal and prominence of genetic drift are more important in shaping local populations of S. paradoxus than fitness differences among genotypes. S. paradoxus may be dispersed by rainwater and by insects such as *Drosophila* (7, 8), from which it has been isolated. Despite these potential dispersal mechanisms, there is genetic differentiation among S. paradoxus populations that is roughly proportional to distance on a scale from centimeters to thousands of kilometers (9). Although population genetic structure and potential dispersal mechanisms have been described, the extent to which wild yeasts actually grow and persist in stasis in their habitats in soil is unknown.

The experimental design for this study included two parts: (i) examination of resident genotypes over time and (ii) the fate of transplanted, marked genotypes. We reasoned that examination of residents could detect persistent and immigrant genotypes, while the transplantation experiments allow assessment of carrying capacity and persistence (even though transplantation experiments cannot detect unmarked genotypes, either resident or immigrant). In the present study, we first monitored naturally occurring genotypes of S. paradoxus over a 3-year period on a fine geographical scale of marked sites in a natural woodland. This extended the time frame covered by our earlier study (10) by 2 years. This part of the study included only enrichment culturing and therefore registered only presence or absence, not abundance, of yeasts. We then initiated transplantation experiments with yeast strains marked with spontaneous *ura3* mutations. The transplanted yeasts in our study allowed quantification of CFU per unit of soil and of change over time from an initially high level of inoculum. We found that abundance of transplanted genotypes fluctuated, with an overall downward trend over time and that rainfall events were associated with a temporary increase in abundance. Even when abundance counts approached zero in some transplant sites, enrichment culture invariably recovered the transplanted genotype. Although our experiments focused on S. paradoxus, we show that marked strains of S. cerevisiae, although not among the residents found in this locality, persist in proximity to transplanted S. paradoxus.

## RESULTS AND DISCUSSION

In 2014 and 2015, yeasts were collected by enrichment culture in ethanol-containing medium from 72 sites around the bases of four oak trees and genotyped for single nucleotide polymorphism (SNP) sites across their genomes. A remarkable pattern of persistence of genotypes in their sites of origin was observed over this 1-year time span. In the present study, we sampled these 72 sites again in 2016 and a limited selection of eight sites again in 2017 (Table 1), extending the sampling times of 2014 and 2015 reported by Xia et al. (10) by 2 years.

**TABLE 1:** Identity of yeast strains recovered by enrichment culture from sites near the bases of four oak trees

Site (tree-site- microsite)^a^	Yeast strain/clade^b^ recovered in:			
	2014	2015	2016	2017
1-1-1	WX1/**a**	R1/**a**	DK1/**a**	
1-1-2	WX2/**a**	R2/**a**	DK2/**a**	JBA1/**a**
1-1-3	WX3/**a**	R3/**a**	DK3/**a**	
1-1-4	WX4/**a**	R4/**a**	DK4/**a**	
1-1-5	WX5/**a**	R5/**a**	DK5/**a**	
1-1-6	WX6/**a**	R6/**a**	DK6/**a**	
1-2-1	WX7/**a**	R7/**a**	DK7/**a**	
1-2-2	WX8/**a**	R8/**a**	DK8/**a**	
1-2-3	WX9/**a**	R9/**a**	DK9/**a**	
1-2-4	WX10/**a**	R10/**a**	DK10/**a**	JBA2/**a**
1-2-5	WX11/**a**	R11/**a**	DK11/**a**	
1-2-6	WX12/**a**	R12/**a**	DK12/**a**	
1-3-1	WX13/**a**	R13/**a**	DK13/**a**	
1-3-2	WX14/**a**	R14/**a**	DK14/**a**	
1-3-3	WX15/**a**	R15/**a**	DK15/**a**	
1-3-4	WX16/**a**	R16/**a**	DK16/**a**	
1-3-5	WX17/**a**	R17/**a**	DK17/**a**	
1-3-6	WX18/**a**	R18/**a**	DK18/**a**	JBA3/**a**

2-1-1	−	−	−	
2-1-2	−	R19/**d**	−	
2-1-3	−	−	−	
2-1-4	WX19/**d** and WX20/**d**	−	−	
2-1-5	−	R20/**d**	−	
2-1-6	−	R21/**d**	−	
2-2-1	−	−	−	
2-2-2	−	R22/**d**	−	
2-2-3	WX21/**d**	R23/**d**	DK19/Pm	
2-2-4	−	R24/**d**	−	
2-2-5	−	−	−	
2-2-6	WX22/**b**	−	−	
2-3-1	−	−	DK20/**e**	
2-3-2	−	Lt	DK21/**e**	
2-3-3	−	−	DK22/**d**	
2-3-4	−	Td	DK23/**d**	
2-3-5	Lt	−	−	
2-3-6	−	Td	−	

3-1-1	−	R28/**a**	DK24/Pk	−
3-1-2	−	R29/**c**	DK25/**c**	
3-1-3	−	−	DK26/**c**	
3-1-4	WX25/**a**	Td	DK27/**c**	
3-1-5	−	−	DK28/**c**	
3-1-6	−	R31/**c**	DK29/**c**	
3-2-1	−	−	−	
3-2-2	−	−	−	
3-2-3	−	−	DK30/**c**	
3-2-4	WX26/**b**	−	−	
3-2-5	WX27/**c**	−	−	
3-2-6	−	−	−	
3-3-1	−	−	−	
3-3-2	−	R32/**c**	DK31/**c**	−
3-3-3	−	−	DK32/**c**	
3-3-4	WX28/**c**	R33/**c**	DK33/**c**	
3-3-5	−	−	DK34/**c**	
3-3-6	WX29/**c**	R34/**c**	−	

4-1-1	WX30/**c**	R35/**a**	−	
4-1-2	WX31/**c**	R36/**c**	−	
4-1-3	WX32/**c**	R37/**c**	DK36/**a**	−
4-1-4	WX33/**c**	R38/**c**	DK37/**c**	
4-1-5	−	−	DK38/**c**	
4-1-6	WX34/**c**	R39/**c**	DK39/**c**	
4-2-1	WX35/**c**	R40/**c**	DK40/**c**	
4-2-2	WX36/**c**	R41/**c**	DK41/**c**	JBA4/**c**
4-2-3	WX37/**c**	R42/**c**	DK42/**c**	
4-2-4	WX38/**c**	R43/**c**	DK43/**c**	
4-2-5	WX39/**c**	R44/**c**	DK44/Pm	
4-2-6	WX40/**c**	R45/**c**	DK45/**c**	
4-3-1	−	−	DK46/**c**	
4-3-2	WX41/**c**	−	DK47/**c**	
4-3-3	WX42/**a**	−	DK48/**c**	
4-3-4	WX43/**c**	R46/**c**	DK49/**c**	−
4-3-5	−	R47/**c**	DK50/**c**	
4-3-6	WX44/**c**	Cc	DK51/**c**	

a Sites are indicated in the form tree-site-microsite. Four oak trees were sampled, with three sites around the base of each tree separated by buttress roots and six microsites in each site.

b Strains are represented as strain name/clade membership (clades shown in boldface type). The presence of a minus symbol indicates that a sample was taken, but no yeast was recovered. In 2014, 2015, and 2016, all 72 oak microsites were sampled; in 2017, eight samples were taken. The data for 2014 and 2015 are from the report of Xia et al. (10), and the data from 2016 and 2017 are from this study. Abbreviations for non-*Saccharomyces* species names: Cc, Candida californica; Lt, Lachancea thermotolerans; Pk, Pichia kudriavzevii; Pm, P. mandshurica; Td, Torulaspora delbrueckii.

Around oak 1, all sites were occupied in all years by a single S. paradoxus genotype, clade a. Here, the rate of recovery of S. paradoxus was remarkable in its completeness across all sites. Oak 2 showed an entirely different pattern of occupancy from that of oak 1. Overall, less than half the sites around oak 2 returned yeasts, but those that did included a broad diversity of genotypes, including the following: S. paradoxus clade d, a hybrid lineage between two divergent lineages; an isolate of clade b, also a hybrid, but between more recently diverged lineages (clades a and c); and clade e, a genotype of European origin. The sites around oak 2 also yielded three other yeast species, Lachancea thermotolerans, Torulaspora delbrueckii, and Pichia mandshurica. Oak 2 is also notable for the complete absence of clades a and c among the recovered yeast samples.

Oak 3 and oak 4 presented partial occupancy of sites, with clade c predominant among the positive sites but clades a and b also found. Three additional yeast species were found; these species were Pichia kudriavezeyii, P. mandshurica, and Candida californica, a species reported to be consistently vectored by Drosophila melanogaster (11). The final year of limited sampling yielded one isolate of clade c. Overall, the pattern of persistence is extended at least through 2016 and is consistent with persistence in the limited sampling in 2017.

 The previous resampling experiments examined yeasts in natural sites around the bases of oak trees by enrichment in ethanol-containing medium; this culture method is not quantitative and only registers presence or absence, with some unknown threshold for detecting presence. In studying natural populations of S. paradoxus, there has been a clear need for a method to quantify transplanted yeasts *in situ*. A recently reported method for quantifying yeasts in their natural habitats (4) used digital droplet PCR, which sensitively measures the ratios of the abundance of transplanted genotypes, but not their absolute abundance. In this study, we used strains marked with spontaneous mutations in the *URA3* gene, which conferred a Ura^−^, 5-fluoroorotic acid (5FOA)-resistant phenotype. With this method, absolute abundance (CFU per gram of soil) could be measured in one step by dilution plating on agar medium containing 5FOA, with no interference from the growth of other soil microorganisms.

**FIG 1:**
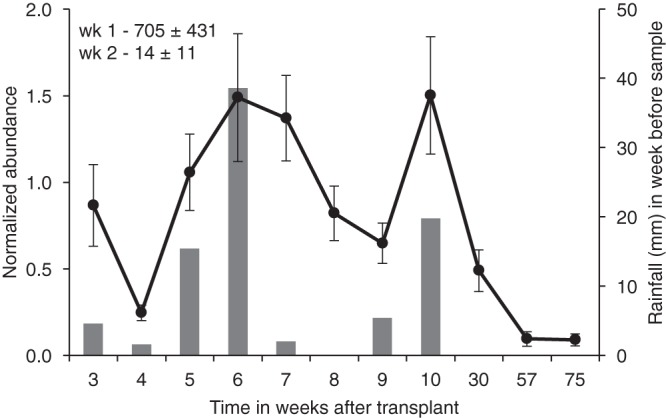
Outdoor transplantation of yeast strains and abundance over time. Three strains were each transplanted to three sites around the bases of three oak trees (three strains per tree) on 23 September 2016. The abundance values for each site were normalized against the average counts of CFU per gram of soil between weeks 3 and 8 ± standard errors (SEs). The average value for normalization (over the nine sites) was 373,440 ± 76,254 CFU/g soil (average ± SE). In the initial sampling, the colonies were too numerous to count on the dilution plates. The average relative abundance values for all nine sites ± standard errors for weeks 1 and 2 appear in the top left corner of the graph, as these values are off the scale of this graph. The bars represent rainfall amount in the week prior to sampling. Rainfall and temperature data are included in Table S2 in the supplemental material.

We transplanted yeast populations of high density into nine sites around the bases of three additional oak trees (oak 6, oak 7, and oak 8) in order to measure their abundance over time in the natural habitat. Figure 1 shows the change in the composite abundance of all transplant populations over time. After a steep decrease in abundance over the first 2 weeks after transplantation, the levels fluctuated over time with seven of nine sites still registering counts one full year after transplantation and two of nine sites returning no CFU. However, enrichment cultures at the end of the experiment, including those two sites registering no CFU in the final plating on 5FOA-containing medium, were all positive for the transplanted strains. The original resident yeasts on these sites sampled before transplantation (see Table S1 in the supplemental material) would not be expected to appear on these plates containing 5FOA; whether or not the original resident strains remain on the transplant sites at low frequency is not known.

10.1128/mSphere.00211-18.1 TABLE S1  Summary of resident yeast species on the sites for outdoor transplantation. Download TABLE S1, XLSX file, 0.03 MB. Copyright © 2018 Anderson et al. 2018 Anderson et al. This content is distributed under the terms of the Creative Commons Attribution 4.0 International license.

**FIG 2:**
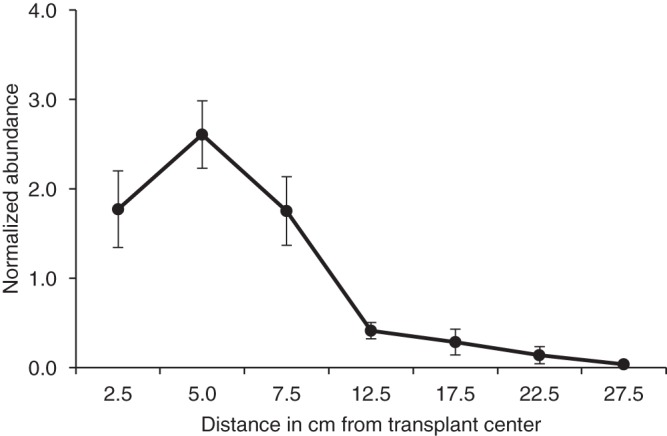
Outdoor transplantation and relative abundance with distance in centimeters from the transplant site. On three of the nine sites for outdoor transplantation (each at a different tree and each a different strain), six diameters were measured at distance intervals (two intervals per site) at week 39 after transplantation on 21 June 2017. The values for each site were normalized against CFU per gram of soil for each of the six diameters ± standard error. The average value for normalization (over the six diameters) was 58,449 ± 14,644 CFU/g soil (average ± SE).

**FIG 3:**
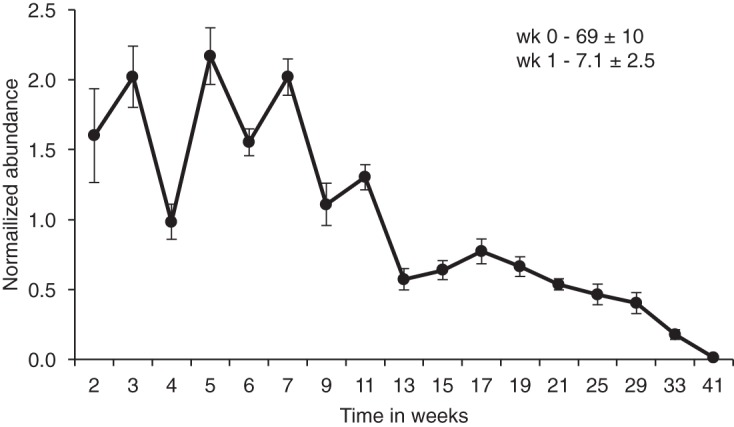
Indoor transplantation of yeast strains and abundance over time. Three strains were transplanted to each of three petri dishes containing 50 g of forest soil (nine plates total) on 12 January 2017. The values for each site were normalized against CFU/gram of soil between weeks 2 and 41 ± standard error. The average value for normalization was 612,118  ± 74,280 CFU/g soil (average ± SE). The values for the initial sampling and week 1 after transplantation appear in the top right corner of the graph.

**FIG 4:**
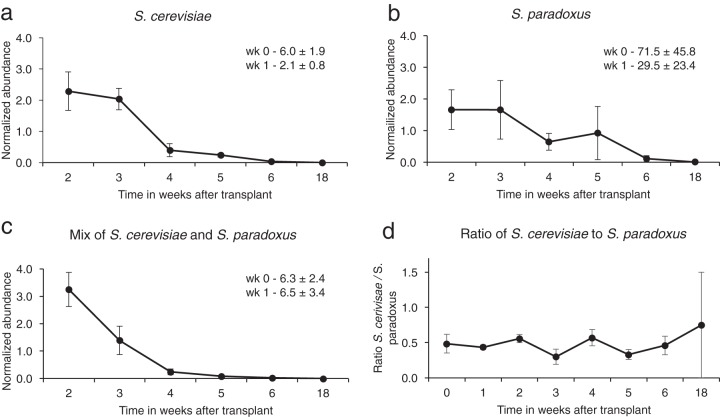
Outdoor transplantation of S. cerevisiae and S. paradoxus and relative abundance over time. (a to c) Inocula containing S. cerevisiae only (a), S. paradoxus only (b), and a mix of S. cerevisiae and S. paradoxus (c) were transplanted to three sites at the bases of three oak trees on 26 October 2017; each tree had one site of each type of inoculum. (d) Ratio of S. cerevisiae and S. paradoxus for the mixed population. Note that the counts on test plates containing 5FOA were low for the 18-week time point at a total of 12 CFU on nine plates. The values for each site were normalized against the values (CFU/gram of soil) between weeks 3 and 10 ± standard error. The average values for normalization were as follows: 738,722 ± 632,755 CFU/g of soil for S. paradoxus, 922,523 ± 592,478 CFU/g of soil for S. cerevisiae, and 1,287,051 ± 315,193 CFU/g of soil for the mix of the two yeasts (average ± SE shown). The average relative abundance values (all nine sites) for weeks 1 and 2, which were off the scale on the graph, appear in the top right corner of each graph.

10.1128/mSphere.00211-18.2 TABLE S2  Raw data (CFU/gram of soil) for Fig. 1 to 4. Download TABLE S2, XLSX file, 0.04 MB. Copyright © 2018 Anderson et al. 2018 Anderson et al. This content is distributed under the terms of the Creative Commons Attribution 4.0 International license.

In addition to abundance, Fig. 1 also indicates rainfall over time. For the first and larger of the two conspicuous rainfall events, between weeks 4 and 6, all nine populations increased in abundance (under the null hypothesis that an increase or decrease in CFU is a random behavior, the probability of the same directionality of response in all nine sites is *P* ≤ 1/2^9^, or 0.002). In the second event, between weeks 8 and 10, eight of the nine populations increased in abundance. At this stage, we interpret the apparent increase in population abundance with rainfall as a possible association, which is addressed below in a laboratory experiment. This transplantation experiment was not expected to distinguish whether the increase was due to reproduction of cells or to separation of aggregated cells because of moisture (driving up CFU on the detection plates) or to some other mechanism.

The outdoor transplants introduced yeast populations to a spot about 5 cm in diameter. At week 39, we recovered samples along six transects extending outward from three transplant sites (Fig. 2). Here the abundance values clearly show that the plume of transplanted yeast cells had spread laterally. How this happened cannot be distinguished in this experiment. The yeast cells may have been washed outward from their central location with rainfall. This lateral spread may in part explain the rapid initial decrease in abundance in the central location after transplantation. Actual reduction in viable cells may also contribute to the reduction in observed CFU. It is also possible that outcrossing between marked strains and residents could reduce apparent counts because the *ura3* allele is recessive and would not be detected when heterozygous with the *URA3*^+^ allele in resident yeasts. Given the generally low outcrossing rate per generation (12), however, we consider it unlikely that outcrossing affects counts substantially.

The next experiment was devised to reproduce the outdoor transplant experiment *in vitro*, with petri dishes containing a limited volume of nonsterile soil. The pattern of abundance over time in the indoor experiment paralleled the outdoor experiment. Yeast abundance initially decreased sharply and then fluctuated over time, gradually approaching low levels by week 41 (Fig. 3). A final enrichment culturing at week 48, followed by testing on 5FOA-containing medium, revealed that all nine plates harbored viable representatives of the originally transplanted strains. Under these conditions, the decline in CFU could not have been due to spreading of the yeast populations as occurred in the outdoor transplantation sites. The decline of CFU must have been due to a decrease in the proportion of viable cells or to an increase in the formation of aggregates (in which multiple viable cells that were aggregated would plate as 1 CFU).

After the indoor transplantation experiment was completed, the soil plates were repurposed for another experiment to test for the effect of moisture after extended drought. The plates were allowed to dry out at room temperature for 3 months. At this point, soil samples were taken for dilution plating on 5FOA-containing medium and for enrichment culture. No CFU were found for this initial sampling; the transplanted yeasts had become rare. Nonetheless, the enrichment cultures were all positive for the transplanted yeasts (which had the 5FOA-resistant phenotype). Water was then added to the plates so that the soil became saturated, but without free water. Additional water was added to the plates twice a week to keep the soil saturated. Samples were taken at week 1 and week 2 after the initial sampling. All plates yielded CFU, and for all replicates, the abundance increased from initial sampling (0.0 ± 0.0 CFU/g of soil) to week 1 (2,247 ± 867 CFU/g of soil) and to week 2 (2,406 ± 802 CFU/g of soil) (means ± standard errors [SEs] shown). The effect in this *in vitro* experiment was remarkably consistent with response to rainfall after a period of dryness in the outdoor transplantation experiment (null hypothesis, an increase or decrease in CFU is random; probability of the same directionality of response in all nine plates is *P* ≤ 1/ 2^9^, or 0.002).

In a final experiment, we addressed the question of whether S. cerevisiae would persist in outdoor transplant sites, as did S. paradoxus. The rationale for this experiment is that S. cerevisiae is not found in our study site or, more generally, in northern latitudes in woodlands (13, 14). Furthermore, genotypes of S. cerevisiae do not appear to spread from vineyards to forests (15). Here, we transplanted a marked genotype of S. cerevisiae in proximity to S. paradoxus. Both yeast species and the mixture declined in abundance over the first 6 weeks to very low CFU counts at the end of the winter (week 18 in Fig. 4). The ratio of the two yeast species fluctuated around 1:1. At the end of the experiment, CFU were low (Table S2); in the mixed population, in total, there were 6 CFU of S. cerevisiae and 6 CFU of S. paradoxus. In enrichment culture at week 19, all nine sites recovered the original genotype transplanted. At this stage, S. cerevisiae appears to persist about as well as S. paradoxus in the study locality, where they have not been detected previously.

There are two main contributions from this study. The first contribution is technical. Although our focus here was on persistence of yeast strains in their habitats, our transplantation experiments demonstrate the feasibility of long-term measurements of fitness effects in mixtures of strains in the field and in the laboratory. An advantage of transplantation to indoor microcosms with nonsterile soil is that the markers used to distinguish strains need not be limited to spontaneous mutations as was the case here for outdoor transplantation experiments. Targeted comparisons might involve specific gene deletions or modifications that would not be appropriate for outdoor transplantation.

The second contribution is biological. Our observations that resident yeast populations persist over time with infrequent dispersal is entirely consistent with the scenario proposed by Boynton et al. (4). The transplantation experiments further suggest that the carrying capacity for yeasts in soil is limited but that yeast populations can nonetheless persist at low levels over the time scale of years. The initial high levels of inoculum in the transplantations were not sustainable. The number of CFU per gram of soil decreased precipitously in the first 2 weeks after transplantation and then more slowly, finally registering few or no CFU on the 5FOA selective medium, and yet, enrichment culturing indicated that the original, transplanted genotypes persisted at their sites in a viable state. Whether or not the transplants actually replace the naturally occurring yeast residents on those sites cannot be assessed with the *ura3* marker system. Also, it remains unknown whether the persisting strains exist in a sporulated or vegetative state; because the persisting yeast cells exist at such low abundances, it is not possible to actually see them in soils. Regardless of the status of the viable propagules, this study did not reach the limits of persistence, which can be found only by sampling the transplant sites in subsequent years.

Why *Saccharomyces* yeasts are found in soil at all is a mystery, given their apparent adaptation to a high-sugar environment. At present, the capacity of yeast populations for growth in soil remains unproven. Therefore, whether soils represent an ecological sink or source for yeast populations is not known. Nonetheless, our results are consistent with the idea that *Saccharomyces* yeasts exist at extremely low levels in the soil, with the possibility for limited population growth when temperature and moisture conditions in the soil permit. Against this prevalent pattern of stasis, the sudden availability of higher nutrient conditions could lead to substantial growth in yeast populations with subsequent decrease in population density over time; our transplantation experiments were designed to simulate these high-growth events. Such high-growth sources might include plant exudates, leaf surfaces, or insect nests, with yeast lineages persisting in soils after wash-off during rains. The overall downward trend of density during the experiment (as shown in the figures) is consistent with this possibility. We speculate that such chance amplifications in yeast populations would have more effect on long-term presence than fitness differences among genotypes, especially where those fitness differences relate to slight differences in growth rate measured under rich nutrient conditions in laboratory culture.

## MATERIALS AND METHODS

### Enrichment recovery of naturally occurring yeasts.

Our initial goal was to identify naturally occurring genotypes of S. paradoxus and to track their distributions over time. The sampling area included the same sites studied via enrichment culture by Xia et al. (10) in 2014 and 2015, with the present study extending the earlier sampling into 2016 and 2017 (Table 1). Sampling was performed around four oak trees, with three sites around the base of each tree separated by buttress roots, with six microsites in each site (Fig. 1) (see Xia et al. [10]). Collection of soil samples, enrichment culture with 8% ethanol, DNA isolation, Illumina sequencing, read alignment against a standard genome, and variant discovery were conducted exactly as described by Xia et al. (10). Each newly isolated strain was identified to a clade (clades a to e); the procedure was described earlier by Xia et al. (10).

### Marked strains for transplantation experiments.

Transplantation experiments were done with spontaneously arising *ura3* mutants. In this study, we did not consider it essential for the marker mutation to be neutral in its fitness effect. *In vitro*, the *ura3* markers impose a slight growth deficit (<1% increase in doubling time relative to the wild type) in rich medium at 30°C. Despite the possible handicap imposed by the *ura3* mutations to detecting persistence in soils, however, the signal of persistence was strong.

We found *ura3* mutants in S. paradoxus by allowing a diploid culture to sporulate and then plating large numbers of spores in tetrads on 5FOA-containing medium. The sporulation step was done to facilitate homozygosity for any newly arising mutants. The resistant mutants appearing on 5FOA-containig medium had a Ura^−^ phenotype (16). Three *ura3* mutants of S. paradoxus were used: one frameshift (Sce5003, c.260_261insC), one nonsense (Sce5006, c.553C > T, GLU to stop), and one missense mutation (Sce5010, c. 801G > A, Gly to Asp).

For a marked version of S. cerevisiae, we crossed a wild-type strain (laboratory no. Sce3, *MAT*α, a recombinant offspring of a cross between ATCC *MAT***a** *leu2*Δ*1* and ATCC *MAT*α *ura3-52*) and another strain (laboratory no. Sce695, open biosystems BY4741, *MAT***a** *his3*Δ*1 leu2*Δ*0 met1*Δ*0 ura3*Δ*0*). From the offspring of this cross, we identified haploid strains of complementary mating type that were 5FOA resistant (and Ura^−^) and His^−^. These two haploids were then mated to form a diploid that was homozygous for *ura3* and *his3*. The His^−^ phenotype was used to distinguish S. cerevisiae from S. paradoxus (His^+^) in mixed transplant sites. None of the transplanted strains of either S. paradoxus or S. cerevisiae contained any genetically engineered or recombinant DNA constructs.

### Outdoor transplantation of S. paradoxus.

Oak 6, oak 7, and oak 8 were selected for transplantation. At each tree, there were three transplant sites separated by buttress roots (total of nine sites). The three sites at each tree were each inoculated with a different strain of the three *ura3* marked strains of S. paradoxus (23 September 2016). Immediately before transplantation, any yeast residents were identified by enrichment culture as described above (see Table S1 in the supplemental material).

At each site, we applied 100 ml of water with a suspension of 10^10^ cells through the perforated cap of a large saltshaker. A spot on the soil surface of about 5 cm in diameter was sprinkled with the cell suspension, allowing sufficient time for the fluid to soak in without running across the surface. At the time of sampling, ca. 0.5 g of soil was scraped into a 15-ml plastic tube, weighed, and suspended in either 10 ml of water or, at the end of the experiment, in 10 ml of enrichment medium (6). After vigorous agitation, 100 µl of the suspension, or a dilution thereof, was spread onto 9-cm petri dishes with 5FOA-containing medium (15). After 3 days of incubation at 30°C, colonies were counted, and the number of CFU per gram of soil was calculated. Source data for transplantation experiments are provided in Table S2. At the end of the experiment and after enrichment culturing, colonies were tested for 5FOA resistance and the Ura^−^ phenotype. Sites without transplantation invariably registered no 5FOA-resistant cultures.

### Indoor transplantation of S. paradoxus.

These experiments were constructed to mimic the outdoor experiments, except that the available volume was limited and the inoculum was initially mixed into the nonsterile soil, which had been collected near the bases of several oak trees, pooled, and sieved to a fine particle size. Deep petri dishes (2.5 by 9 cm) containing ca. 50 g soil mix were inoculated with 10 ml of water containing a suspension of 10^9^ cells (12 January 2017). Each soil plate was weighed initially and then weekly thereafter; the reduction in weight over the preceding week was compensated for by the addition of sterile water. After this experiment was completed, the soil plates were repurposed to test the effect of moisture addition after a period of extended dryness as described below. The plates were allowed to dry out at room temperature (RT) for 3 months, and CFU were measured before and after the addition of water to the plates.

### Outdoor transplantation of S. cerevisiae.

We selected three new trees (oak 9, oak 10, and oak 11) with three sites around the base of each tree separated from one another by buttress roots. At each tree, S. cerevisiae was transplanted to one site, S. paradoxus to another site, and an equal mix of the two species to a third site. Transplantation of yeast cells was done exactly as in the outdoor transplantation experiment for S. paradoxus***.*** Approximately 10^10^ cells suspended in water were applied to each site (26 October 2017).

### Data availability.

A comprehensive variant (.vcf) file for all S. paradoxus strains in Table 1 is available in the Dryad Digital Repository (doi:10.5061/dryad.2th3266). Alignments of Illumina reads of the 2014 and 2015 collections with a reference S. paradoxus genome (.bam files) are accessible through NCBI’s SRA (https://www.ncbi.nlm.nih.gov/sra) under accession number PRJNA324830 (10).
